# Impaired right atrial function preceding right ventricular systolic dysfunction: clinical utility and long-term prognostic value in pulmonary hypertension

**DOI:** 10.1186/s13244-025-01996-6

**Published:** 2025-06-04

**Authors:** Fan Yang, Yan Yan, Wang Jiang, Zhouming Wang, Caixin Wu, Qian Wu, Yuanlin Deng, Yamin Du, Zhenwen Yang, Zhang Zhang, Dong Li

**Affiliations:** 1 Department of Radiology, Tianjin Medical University General Hospital, Tianjin, China; 2 Department of Radiology, Tianjin Key Lab of Functional Imaging & Tianjin Institute of Radiology, Tianjin Medical University General Hospital, Tianjin, China; 3 Department of Cardiology, Tianjin Medical University General Hospital, Tianjin, China; 4 Department of Radiology, Tangshan Maternal and Child Health Hospital, Hebei, China

**Keywords:** Right atrial function, Pulmonary hypertension, Prognosis, Magnetic resonance imaging, Right ventricular dysfunction

## Abstract

**Objectives:**

Pulmonary hypertension (PH) in patients with right ventricular systolic dysfunction (RVSD) is associated with a poor prognosis. This study assessed the characteristics of right atrial (RA) function using cardiac magnetic resonance feature tracking (CMR-FT) before RVSD onset and evaluated the long-term prognostic significance of these characteristics.

**Materials and methods:**

A total of 96 PH patients, including 36 without RVSD (PH-nonRVSD) and 60 with RVSD (PH-RVSD), were compared to 20 healthy controls (HCs). The RA reservoir, conduit, booster pump functions, and the right ventricular global longitudinal strain (RVGLS) were evaluated. Ventricular morphological and functional parameters of the RA and right ventricle (RV) were also acquired.

**Results:**

Compared with HCs, both RA reservoir and conduit functions were significantly reduced (*p*_*s*_ < 0.05) in the PH-nonRVSD, without significant morphological changes in either the RA or RV (*p*_*s*_ > 0.05). The RA reservoir and conduit function were significantly correlated with the right ventricular ejection fraction (RVEF), RVGLS, pulmonary vascular resistance, brain natriuretic peptide, cardiac index, and 6-min walk distance. Receiver operating characteristic analysis demonstrated that RA conduit function outperformed RVGLS and RVEF in differentiating PH-nonRVSD and HCs. However, a reduction in RA booster pump function was observed only in the PH-RVSD group (*p* < 0.001). During a median follow-up period of 97 (80–106) months, 45% of the included patients died. RA reservoir function was an independent predictor of all-cause mortality (HR = 0.963, 95% CI: 0.935–0.992, *p* = 0.014).

**Conclusions:**

RA function can detect right heart dysfunction prior to RVSD and monitor disease progression in patients with PH. Moreover, RA reservoir function independently predicts long-term prognosis.

**Critical relevance statement:**

Impairment of right atrial (RA) function, assessed by cardiac magnetic resonance feature tracking (CMR-FT), in pulmonary hypertension (PH) patients is sensitive in detecting right-sided heart dysfunction before right ventricular systolic dysfunction and can be utilized to monitor disease progression and long-term prognosis.

**Key Points:**

- RA function is sensitive in detecting early right heart dysfunction in PH patients.
- The disease progression of PH can be monitored by assessing RA function.
- RA function can serve as a tool for predicting long-term prognosis.

**Graphical Abstract:**

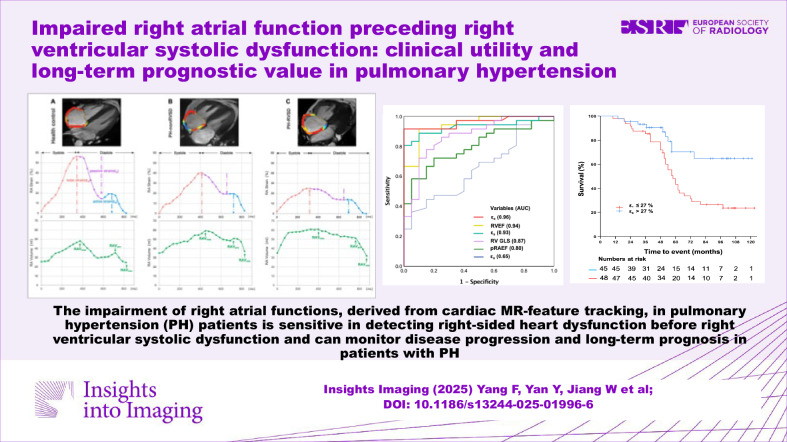

## Introduction

Pulmonary hypertension (PH) is a serious pathophysiological disorder characterized by persistent elevation of pulmonary vascular resistance, progressive right heart dysfunction, and eventual heart failure [1, 2]. Right ventricular systolic dysfunction (RVSD) is commonly observed in PH patients and is associated with more severe clinical features and a poorer prognosis [3–5]. Due to the absence of specific signs and symptoms in the early stages before RVSD develops, PH is typically diagnosed at an advanced stage [6, 7]. Therefore, identifying features of right heart dysfunction prior to RVSD is crucial for implementing appropriate interventions and improving patient outcomes [8].

Right atrium (RA) function can be divided into three phases during the cardiac cycle: reservoir, conduit, and booster pump [9]. The RA function is vital in regulating RV filling and maintaining cardiac output. Assessment of RA function can be derived from RA strain and RA emptying fraction (RAEF). Specifically, evaluation of atrial reservoir function corresponds to RA total strain (ε_s_) and total RAEF (tRAEF). And the assessment of atrial conduit function is represented by RA passive strain (ε_e_) and passive RAEF (pRAEF). Additionally, atrial booster pump function is evaluated by RA active strain (ε_a_) and active RAEF (aRAEF). Cardiac magnetic resonance (CMR) serves as the reference standard for assessing cardiac function [10]. The CMR feature tracking (CMR-FT) technique has been increasingly recognized as a feasible and reproducible method for determining atrial function by automatically tracking cardiac structures to monitor changes in myocardial strain. Previous studies have demonstrated an association between RA function and alterations in RV function in patients with PH [11, 12]. However, it remains unclear whether changes in RA function occur during the early stage of precapillary PH before RVSD development. Therefore, exploring the characteristics of RA function may provide additional valuable information when evaluating PH, particularly for early detection purposes. We hypothesized that impairment of RA function would manifest at an early stage of precapillary PH before RVSD develops. Furthermore, we investigated the characteristics of RA function in patients without RVSD, observed its evolution during disease progression with RVSD, and assessed its prognostic value in precapillary PH.

## Materials and methods

### Study participants

A retrospective study was conducted on precapillary PH patients who underwent right heart catheterization (RHC) and CMR examinations from January 2013 to May 2021. Inclusion criteria were as follows: (1) age was 18 years or older; (2) RHC results meeting the precapillary PH diagnostic criteria [13], defined as an increase in mean pulmonary arterial pressure (mPAP) to > 20 mmHg with pulmonary arterial wedge pressure (PAWP) ≤ 15 mmHg and pulmonary vascular resistance (PVR) > 2 Wood units according to the guidelines of the European Society of Cardiology (ESC) and the European Respiratory Society (ERS); (3) the interval between RHC and CMR examinations was less than 2 weeks; and (4) patients had not received any treatment by completion of RHC and CMR. Exclusion criteria included the presence of congenital heart disease, severe valvular heart disease, myocardial infarction, cardiomyopathy, atrial fibrillation or flutter, or poor CMR image quality that was unsuitable for post-processing analysis. Patients with precapillary PH were divided into two groups based on the right ventricular ejection fraction (RVEF) derived from CMR: (1) PH without RVSD (PH-nonRVSD): RVEF ≥ 45%; (2) PH with RVSD (PH-RVSD): RVEF < 45% [4, 5]. In addition, 20 healthy volunteers were recruited from the community as healthy controls (HCs). These individuals underwent clinical evaluation (clinical assessment, laboratory testing, and imaging) to confirm the absence of any clinically recognized cardiovascular diseases, cardiovascular risk factors (hypertension, diabetes mellitus, hyperlipidaemia, and renal dysfunction), or respiratory diseases. None of the HCs were taking any medications. All patients received standardized treatment for precapillary PH according to current guidelines after diagnosis. The study was approved by the hospital’s Ethics Committee and conducted in compliance with all the clinical practice requirements as prescribed by the committee. Written informed consent was obtained from all participants, including CMR image analysis.

### Right heart catheterization

All patients with precapillary PH underwent RHC. The following parameters were measured and calculated: mPAP, PVR, PAWP, mean right atrial pressure (mRAP), and cardiac index (CI).

### CMR data acquisition and post-processing

CMR acquisition was performed using a 3.0-T MR scanner, either the Magnetom Skyra (Siemens Medical Solutions) or the MR 750 (GE Healthcare). Standard cine images in 4-chamber and short-axis views were acquired using a cardiac-gated balanced steady-state free precession sequence. Typical parameters are provided in the Supplementary material.

Image analysis was performed using dedicated software (CVI42, version 5.16.0; Circle Cardiovascular Imaging, Inc.). The RA maximal volume (RAV_max_) at RV end-systole, RA pre-emptying volume (RAV_pre_) at RV diastole before RA contraction, and RA minimal volume (RAV_min_) at RV end-diastole were measured from four-chamber cine images by the area-length method [14]. RA areas on the corresponding frame were automatically tracked in the 4-chamber view. In addition, the length from the midpoint between the septal and lateral insertion of the tricuspid valve to the roof of the RA was measured on corresponding frames. RA volumes were then automatically calculated using the area-length method for ellipsoid bodies as previously described [14]. RAEF was calculated as the fractional volume change, including tRAEF, pRAEF, and aRAEF [15]. Morphological parameters of the RV and left ventricle (LV) were automatically tracked on short-axis stack cine images and standardized by body surface area (BSA) as the end-diastole volume index (EDVi), end-systole volume index (ESVi), and stroke volume index (SVi). All automated tracking outputs were reviewed and manually adjusted if needed.

**Fig. 1:**
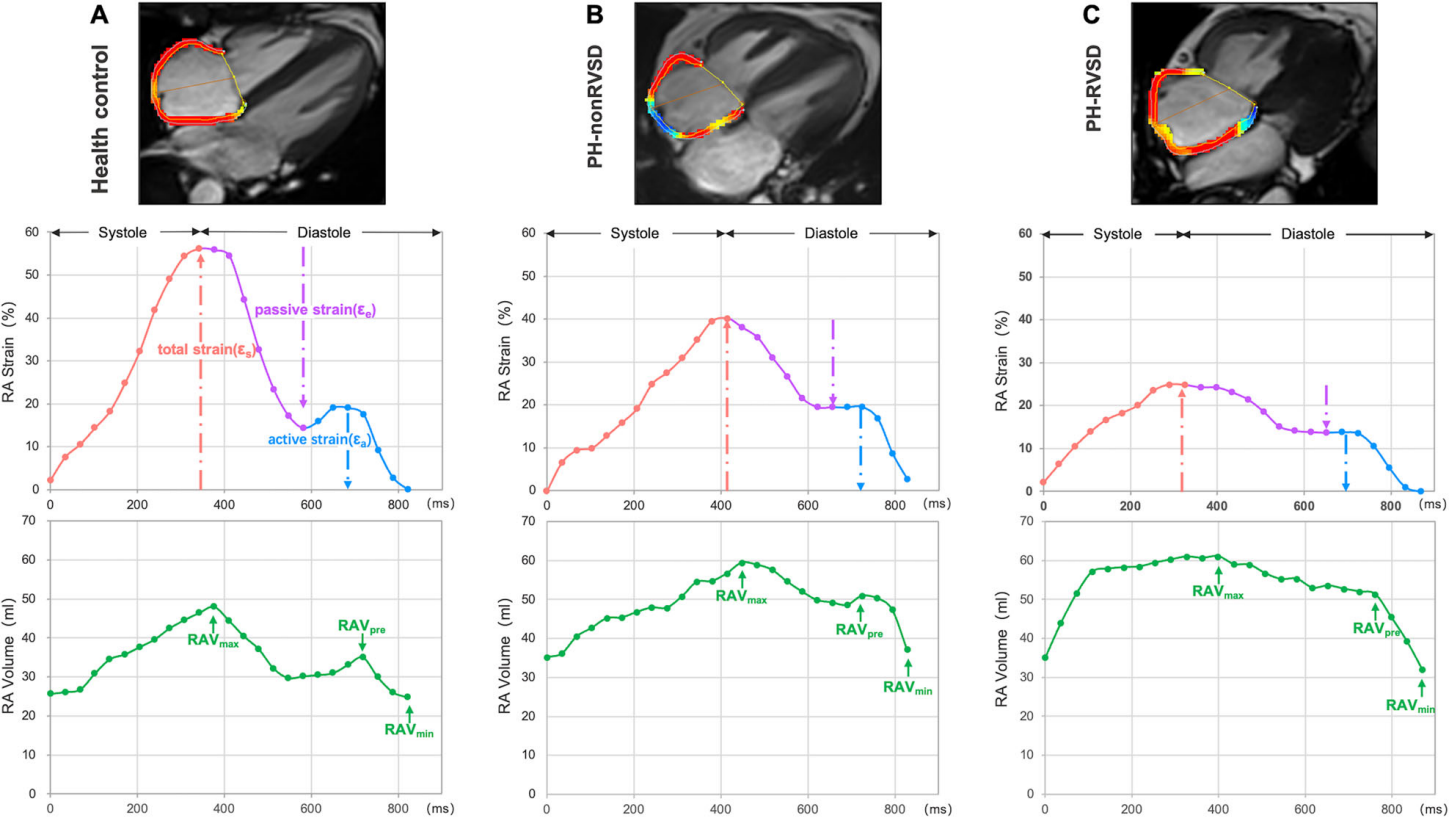
Representative examples of right atrial (RA) contours drawn at right ventricle end-systole (top) and the corresponding RA strain curves (medium) and RA volume curves (bottom) throughout the cardiac cycle in (**A**) a healthy control, (**B**) a PH patient without right ventricular systolic dysfunction (PH-nonRVSD), and (**C**) a PH patient with right ventricular systolic dysfunction (PH-RVSD). RAVmax, RA maximal volume; RAVpre, RA pre-emptying volume; RAVmin, RA minimal volume; ε_s_, total strain; ε_e_, passive strain; ε_a_, active strain

The RA and RV strains were analyzed from four-chamber cine images (Fig. 1). The following strain parameters were derived: RAε_s_ and total strain rate (SR_s_), RAε_e_ and passive strain rate (SR_e_), RAε_a_ and active strain rate (SR_a_), and RV global longitudinal peak strain (GLS). RAε_s_ represents the total deformation of the right atrium during the entire cardiac cycle (from filling to contraction). RAε_e_ reflects passive atrial stretching as blood flows into it during early ventricular relaxation. RAε_a_ captures the atrium’s active contraction to push blood into the right ventricle. RV GLS quantifies how much the RV muscle shortens along its length during contraction. A lower (more negative) RV GLS indicates stronger contraction (e.g., −20% is better than −10%). Assessment of interobserver variability for strain parameters was performed on 25 randomly selected subjects (10 HCs and 15 PH patients) by two experienced radiologists. Intraobserver reproducibility was evaluated by one observer who repeated the measurements 4 weeks later. CMR analysis was performed by readers completely blinded to the clinical data.

### Outcomes

The primary outcome was all-cause mortality after reviewing hospital records or telephone follow-ups with patients or their families by an investigator blinded to the clinical and CMR imaging data. Patients were observed until December 2023. The interval between the date of CMR imaging and death was defined as the time to event.

### Statistical analysis

Statistical analysis was performed using IBM SPSS Statistics software (version 27.0) and GraphPad Prism (version 9.5.0). Continuous variables with a normal distribution and homogeneity of variance are presented as means ± standard deviations; otherwise, they are presented as medians with interquartile ranges. The two PH groups were compared using an independent-sample *t*-test or Mann‒Whitney *U* test. One-way ANOVA or the Kruskal‒Wallis *H* test was used to compare among the three groups, and the Bonferroni correction was applied for post hoc pairwise comparisons. Categorical variables are presented as counts and percentages and were compared using Fisher’s exact test. Correlations between continuous variables were evaluated using the Pearson correlation coefficient or the Spearman rank correlation coefficient. Receiver operating characteristic (ROC) analysis was performed on the RA and RV functional parameters to evaluate their prediction efficiency for PH-nonRVSD. Reader reproducibility was assessed using the intraclass correlation coefficient (ICC) and Bland‒Altman analysis. Cox regression analysis was used for survival analyses in all PH patients; associated parameters (*p* < 0.05) in univariate Cox regression were entered into the multivariate regression analysis using forward stepwise selection. Kaplan–Meier survival curves were constructed to assess the event-free survival curves, and the event rates were compared using the log-rank test. *p* < 0.05 was considered a statistically significant difference.

## Results

### Patient characteristics

**Fig. 2:**
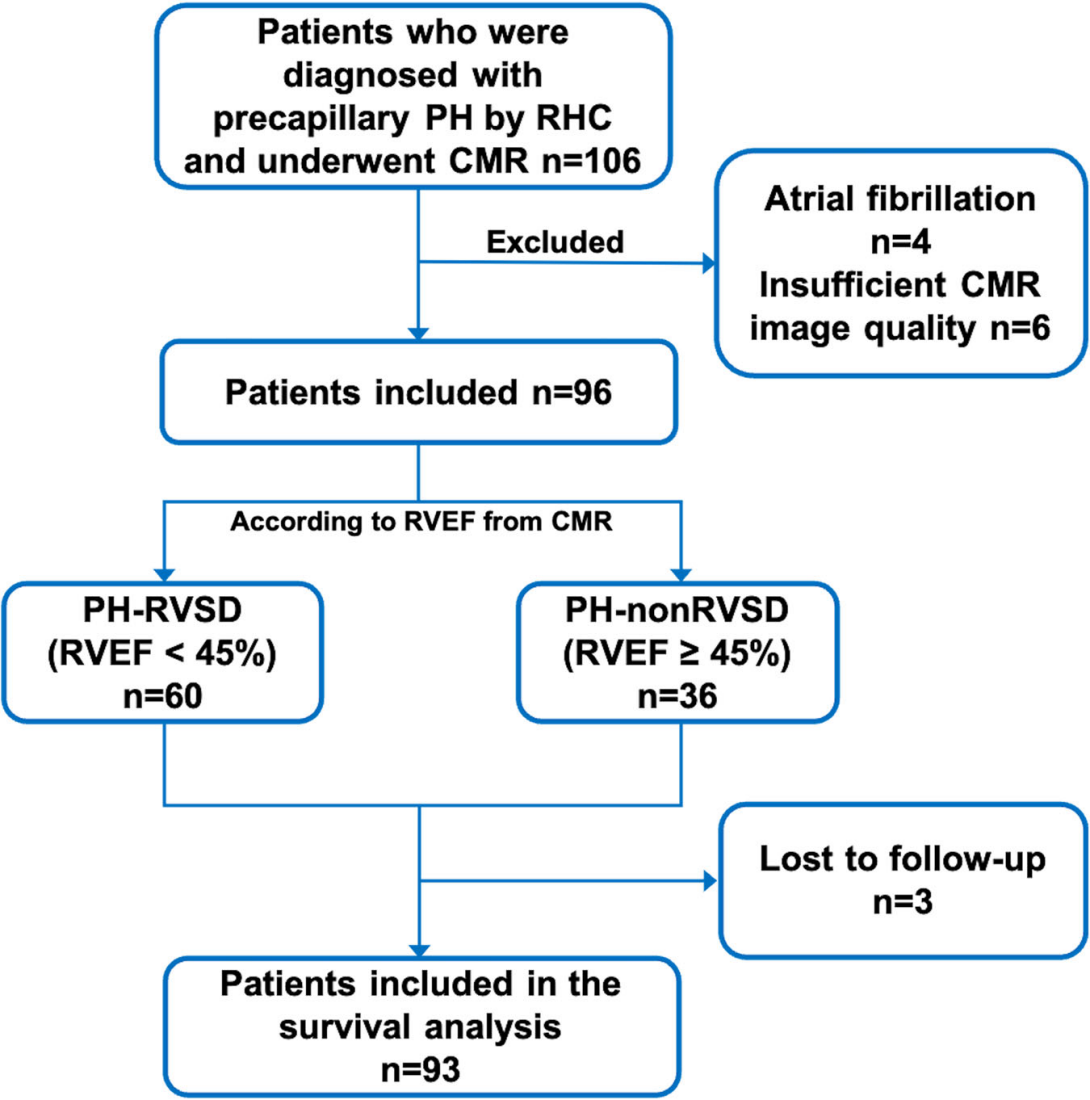
Patient flowchart. PH, pulmonary hypertension; CMR, cardiac magnetic resonance; RHC, right heart catheterization; RVEF, right ventricular ejection fraction; PH-nonRVSD, pulmonary hypertension patients without right ventricular systolic dysfunction; PH-RVSD, pulmonary hypertension patients with right ventricular systolic dysfunction

A total of 106 precapillary PH patients confirmed by RHC were included. Of these, four individuals were excluded due to atrial fibrillation, and six due to insufficient CMR image quality (Fig. 2). Among the included patients, 68 had pulmonary arterial hypertension (PAH), and 28 had chronic thromboembolic pulmonary hypertension (CTEPH). The final analysis cohort comprised 36 PH-nonRVSD patients, 60 PH-RVSD patients, and 20 HCs. The interval between RHC and CMR was 6 (1–12) days. Patients in the PH-nonRVSD group were significantly older than those in the PH-RVSD group (*p* = 0.005). PH-RVSD patients presented with higher BNP serum levels (*p* < 0.001) and a shorter 6-min walk distance (6MWD) (*p* = 0.049). Among the RHC parameters, the PH-RVSD group demonstrated a significantly lower CI (*p* = 0.004) and higher PVR (*p* < 0.001) than the PH-nonRVSD (Table 1).

**Table 1:** Patient characteristics and right heart catheterization (RHC) parameters of subjects

Variables	Healthy controls (*n* = 20)	PH-nonRVSD (*n* = 36)	PH-RVSD (*n* = 60)	*p*-value
Age (years old)	41 (32–57)	59 (37–64)	40 (30–55)	0.007
Female (%)	16 (80)	33 (92)	51 (85)	0.513
BSA (m^2^)	1.6 ± 0.1	1.6 ± 0.2	1.6 ± 0.2	0.810
WHO FC III-IV	-	14 (39)	27 (45)	0.671
6MWD (m)	-	373 (263–442)	273 (157–404)	0.049
BNP (pg/mL)	-	67 (24–309)	342 (132–748)	< 0.001
RHC				
mPAP (mmHg)	-	43 ± 11	51 ± 12	0.002
PVR (Wood unit)	-	9 (7–14)	14 (11–16)	< 0.001
PAWP (mmHg)	-	9 (7–11)	9 (6–11)	0.627
CI (L/min per m^2^)	-	2.7 ± 0.9	2.2 ± 0.7	0.004
mRAP (mmHg)	-	5 (4–8)	6 (4–9)	0.571
PAH (%)		24 (67)	44 (73)	0.487
CTEPH (%)		12 (33)	16 (27)	
Comorbidities				
Hypertension (%)		9 (25)	7 (12)	0.090
Diabetes mellitus (%)		4 (11)	4 (7)	0.468
Treatments				
Mono-therapy (%)		21 (58)	32 (53)	0.556
Combination therapy (%)		9 (25)	21 (35)	
BPA (%)		12 (33)	16 (27)	0.487

*RHC* right heart catheterization, *PH-nonRVSD* pulmonary hypertension patients without right ventricular systolic dysfunction, *PH-RVSD* pulmonary hypertension patients with right ventricular systolic dysfunction, *BSA* body surface area, *WHO FC* World Health Organization functional classification, *6MWD* 6-min walk distance, *BNP* brain natriuretic peptide, *mPAP* mean pulmonary arterial pressure, *PVR* pulmonary vascular resistance, *PAWP* pulmonary arterial wedge pressure, *CI* cardiac index, *mRAP* mean right atrial pressure, *PAH* pulmonary arterial hypertension, *CTEPH* chronic thromboembolic pulmonary hypertension, *BPA* balloon pulmonary angioplasty

### RV and LV characteristics

Compared with HCs, the RVEF (*p* = 0.024) and RV GLS (*p* = 0.008) were lower in PH-nonRVSD patients, whereas the other parameters were not significantly different. The RVEF (*p* < 0.001) and RV GLS (*p* < 0.001) were further decreased in the PH-RVSD. Compared with the PH-nonRVSD, the PH-RVSD had significantly greater RVEDVi and RVESVi values and lower RVSVi values (Table 2).

**Table 2:** Right ventricle (RV) and left ventricle (LV) characteristics of healthy controls and PH patients

Variables	Healthy controls (*n* = 20)	PH-nonRVSD (*n* = 36)	PH-RVSD (*n* = 60)	*p*-value
RVEDVi (mL/m^2^)	75 (66–84)	81 (67–95)	117 (97–145)***^†^^†^^†^	< 0.001
RVESVi (mL/m^2^)	33 (25–37)	41 (32–50)	88 (66–105)***^†^^†^^†^	< 0.001
RVSVi (mL/m^2^)	44 ± 6	42 ± 10	33 ± 11***^†^^†^	0.02
RVEF (%)	58 (55–63)	48 (46–54)*	26 (23–33)***^†^^†^^†^	< 0.001
RV GLS (%)	−24 (−27 to −23)	−20 (−23 to −17)**	−13 (−16 to −11)***^†^^†^^†^	< 0.001
LVEDVi (mL/m^2^)	68 (62–74)	70 (60–80)	64 (49–75)	0.151
LVESVi (mL/m^2^)	30 (22–34)	27 (20–41)	26 (20–38)	0.997
LVSVi (mL/m^2^)	39 (37–42)	44 (30–47)	34 (26–42)*^†^^†^	0.009
LVEF (%)	58 ± 6	58 ± 10	53 ± 11	0.024

*PH* pulmonary hypertension, *PH-nonRVSD* pulmonary hypertension patients without right ventricular systolic dysfunction, *PH-RVSD* pulmonary hypertension patients with right ventricular systolic dysfunction, *RVEDVi* right ventricular end-diastolic volume index, *RVESVi* right ventricular end-systolic volume index, *RVSVi* right ventricular stroke volume index, *RVEF* right ventricular ejection fraction, *RV GLS* right ventricular global longitudinal peak strain, *LVEDVi* left ventricular end-diastolic volume index, *LVESVi* left ventricular end-systolic volume index, *LVSVi* left ventricular stroke volume index, *LVEF* left ventricular ejection fraction

* *p* < 0.05, ** *p* < 0.01, *** *p* < 0.001 versus the health controls; ^†^^†^ *p* < 0.01, ^†^^†^^†^ *p* < 0.001 versus the PH-nonRVSD

### RA characteristics

Among the RA volume indices (Tables 3, S1), RAV_max_, RAV_pre_, and RAV_min_ were significantly larger in the PH-RVSD group than in the PH-nonRVSD group and HCs (all *p* < 0.05). Concerning RA reservoir function, ε_s_ and SR_s_ were significantly lower in PH-nonRVSD patients than in HCs (*p* < 0.001). In addition, ε_s_ in the PH-RVSD group was lower than in the PH-nonRVSD group (*p* < 0.001). RA’s ability to expand and store blood declines as PH progresses, especially when RV dysfunction develops. However, there was no difference in tRAEF between PH-RVSD patients and HCs (*p* > 0.05). Concerning RA conduit function, ε_e_, SR_e_, and pRAEF were significantly reduced in the PH-nonRVSD and further reductions in the PH-RVSD (all *p* < 0.05). The RA is impaired in passively delivering blood to the RV during early filling, especially in PH-RVSD. Concerning the RA booster pump function, ε_a_, SR_a_, and aRAEF were not significantly different between the HCs and PH-nonRVSD patients. However, compared with the PH-nonRVSD, the PH-RVSD had significantly lower ε_a_ and SR_a_ but similar aRAEF values (Table 3). The ability of the RA to actively squeeze blood into the RV is maintained. The intra- and interobserver reliability of the RA strain parameters (strains and strain rates) were good to excellent, except that the interobserver ICC of SR_a_ was 0.73 (see Fig. S1 and Table S2 in the Supplementary material).

**Table 3:** Right atrium (RA) characteristics of healthy controls and PH patients

Variables	Healthy controls (*n* = 20)	PH-nonRVSD (*n* = 36)	PH-RVSD (*n* = 60)	*p*-value
RA strain (%)				
ε_s_	56 (49–62)	34 (28–43)**	21 (14–29)***^†^^†^^†^	< 0.001
ε_e_	32 (29–38)	16 (12–21)***	10 (7–16)***^†^^†^	< 0.001
ε_a_	22 ± 6	19 ± 7	10 ± 7***^†^^†^^†^	< 0.001
RA strain rate (s^−^^1^)				
SR_s_	2.4 (2.2–2.8)	1.7 (1.1–2.2)**	1.1 (0.8–1.4)***^†^^†^	< 0.001
SR_e_	−2.6 (−3.1 to −2.2)	−1.3 (−1.7 to −0.8)***	−0.7 (−1.0 to −0.5)***^†^^†^	< 0.001
SR_a_	−2.6 (−3.2 to −2.1)	−2.2 (−2.7 to −1.6)	−1.4 (−1.7 to −0.9)***^†^^†^^†^	< 0.001
RA volume index (mL/m^2^)				
RAV_max_ index	36 (30–41)	39 (32–48)	48 (36–74)**^†^	0.018
RAV_pre_ index	25 (21–33)	31 (24–41)	45 (33–68)***^†^^†^	< 0.001
RAV_min_ index	16 (13–21)	18 (15–27)	29 (20–42)***^†^^†^	< 0.001
RA emptying fraction (%)				
tRAEF	54 ± 7	46 ± 13	40 ± 12***	< 0.001
pRAEF	26 ± 8	15 ± 11***	11 ± 8***^†^	< 0.001
aRAEF	37 ± 8	36 ± 13	33 ± 13	0.43

*RA* right atrial, *PH-nonRVSD* pulmonary hypertension patients without right ventricular systolic dysfunction, *PH-RVSD* pulmonary hypertension patients with right ventricular systolic dysfunction, *ε*_*s*_ total strain, *ε*_*e*_ passive strain, *ε*_*a*_ active strain, *SR*_*s*_ total strain rate, *SR*_*e*_ passive strain rate, *SR*_*a*_ active strain rate, *RAV*_*max*_ RA maximal volume, *RAV*_*pre*_ RA pre-emptying volume, *RAV*_*min*_ RA minimal volume, *tRAEF* total RA emptying fraction, *pRAEF* passive RA emptying fraction, *aRAEF* active RA emptying fraction

** *p* < 0.01, *** *p* < 0.01 versus the health controls; ^†^ *p* < 0.05, ^†^^†^ *p* < 0.01, ^†^^†^^†^ *p* < 0.001 versus the PH-nonRVSD

### Correlations of RA strain with RV function, RHC, and risk stratification parameters

Among all subjects, ε_s_, ε_e_, and ε_a_ were significantly correlated with RV GLS (*r* = −0.78, −0.64, and −0.69, respectively; all *p* < 0.001) and RVEF (*r* = 0.79, 0.66, and 0.69, respectively; all *p* < 0.001). Regarding the correlations between RA strain and RHC parameters, ε_s_, ε_e_, and ε_a_ were negatively correlated with PVR and positively correlated with CI. ε_s_ and ε_a_ were negatively correlated with mPAP. However, ε_s_, ε_e_ and ε_a_ did not significantly correlate with mRAP (*p* > 0.05). ε_s_, ε_e_ and ε_a_ had positive correlations with the 6MWD (*r* = 0.37, 0.21, and 0.42, respectively) and negative correlations with BNP (*r* = −0.51, −0.29, and −0.55, respectively). ε_s_ and ε_e_ had a mild correlation with WHO FC (Table 4).

**Table 4:** The correlations of right atrium (RA) strain with right ventricle (RV) function, right heart catheterization (RHC), and risk stratification parameters

Variables	ε_s_		ε_e_		ε_a_	
	*r*-value	*p*-value	*r*-value	*p*-value	*r*-value	*p*-value
RV function						
RVEF	0.79	< 0.001	0.66	< 0.001	0.69	< 0.001
RV GLS	−0.78	< 0.001	−0.64	< 0.001	−0.69	< 0.001
RHC						
mPAP	−0.28	0.007	-	-	−0.30	0.003
PVR	−0.50	< 0.001	−0.28	0.006	−0.54	< 0.001
CI	0.47	< 0.001	0.25	0.015	0.50	< 0.001
WHO FC	−0.21	0.04	−0.238	0.02	-	-
6MWD	0.37	< 0.001	0.21	0.037	0.42	< 0.001
BNP	−0.51	< 0.001	−0.29	0.005	−0.55	< 0.001

*ε*_*s*_ total strain, *ε*_*e*_ passive strain, *ε*_*a*_ active strain, *RV* right ventricle, *RVEF* right ventricular ejection fraction, *RV GLS* right ventricular global longitudinal peak strain, *RHC* right heart catheterization, *mPAP* mean pulmonary arterial pressure, *PVR* pulmonary vascular resistance, *CI* cardiac index, *WHO FC* World Health Organization functional classification, *6MWD* 6-min walk distance, *BNP* brain natriuretic peptide

### Detection performance for PH-nonRVSD

**Fig. 3:**
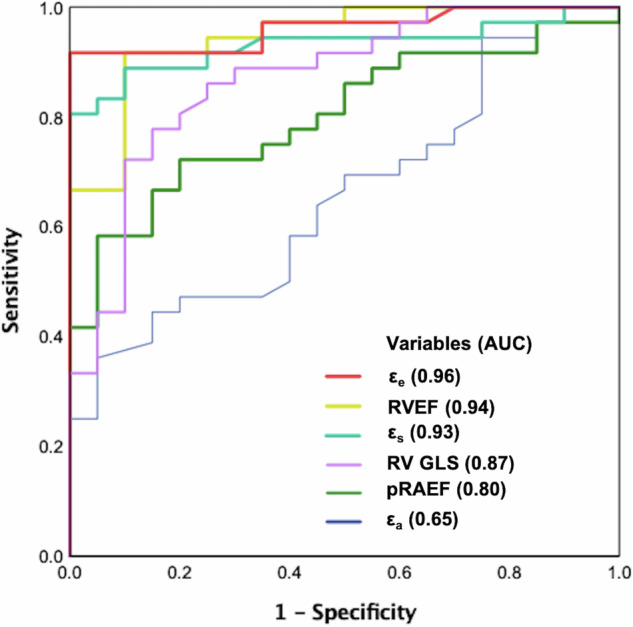
Receiver operating characteristic (ROC) analysis of right atrial and right ventricular functional parameters in differentiating pulmonary hypertension patients without right ventricular systolic dysfunction (PH-nonRVSD) from healthy controls. AUC, area under the curve; CI, confidence interval; RV, right ventricle; RVEF, right ventricular ejection fraction; GLS, global longitudinal peak strain; ε_s_, total strain; ε_e_, passive strain; ε_a_, active strain; pRAEF, passive right atrial emptying fraction

ROC analysis revealed the ability of ε_s_, ε_e_, ε_a_, RV GLS, RVEF, and pRAEF to distinguish PH-nonRVSD patients from HCs (Fig. 3). The areas under the curve for ε_s_, ε_e_, and pRAEF were 0.93 (95% CI: 0.86–1.00, sensitivity: 0.81, specificity: 1.00), 0.96 (95% CI: 0.91–1.00, sensitivity: 0.92, specificity: 1.00), and 0.80 (95% CI: 0.69–0.91, sensitivity: 0.58, specificity: 0.95), with optimal cut-off values of 44%, 26%, and 16%, respectively (all *p* < 0.001). The areas under the curve for RV GLS and RVEF were 0.87 (95% CI: 0.77–0.97, sensitivity: 0.78, specificity: 0.85) and 0.94 (95% CI: 0.89–1.00, sensitivity: 0.92, specificity: 0.90), with optimal cut-off values of −23% and 55%, respectively (all *p* < 0.001). Among the above parameters, ε_e_ presented the highest area under the ROC curve. ε_a_ did not exhibit significant power for predicting PH-nonRVSD (*p* = 0.06) (see Table S3 in the Supplementary material).

### Outcomes

**Fig. 4:**
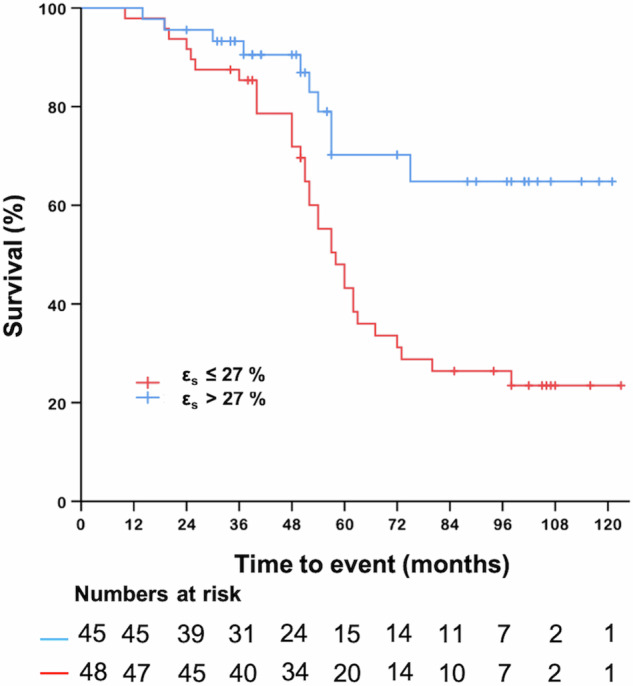
Kaplan–Meier survival analysis for right atrial total strain (ε_s_). Reduced ε_s_ (≤ 27%) was associated with higher rates of all-cause mortality. ε_s_, total strain

During the follow-up period (mean: 82 months, median: 97 months (interquartile range: 50–106 months)), 43 (45%) patients died, and 3 patients were lost to follow-up. The overall survival rates were 98%, 84%, and 49% at 1, 3, and 5 years, respectively. The median survival time was 63 months. Among the patients who died, 32 were PAH patients and 11 were CTEPH patients, and there was no significant difference in mortality between these two aetiologies (*p* = 0.497). Univariate Cox regression analysis revealed that a lower ε_s_, LVEDVi, and LVSVi and a higher SR_a_, RVESVi, mPAP, and PVR were univariate predictors of all-cause mortality in PH patients (*p* < 0.05) (Table 5). By using multivariate stepwise Cox analysis (Table S4), only lower ε_s_ (HR = 0.963, 95% CI: 0.935–0.992, *p* = 0.014) remained strong predictors of mortality, and the cut-off value of ε_s_ was 27%. Kaplan–Meier survival analyses revealed that patients with ε_s_ ≤ 27% had a poor prognosis (log rank, *p* = 0.002) (Fig. 4).

**Table 5:** Predictors of mortality with univariate Cox proportional hazard regression analysis in patients with PH

Variables	Univariate analysis	
	HR (95% CI)	*p*-value
Age (years)	1.018 (0.996–1.041)	0.116
Sex (female)	0.511 (0.214–1.225)	0.132
WHO FC	1.578 (0.966–2.580)	0.069
BNP (pg/mL)	1.000 (1.000–1.001)	0.304
6MWD (m)	0.999 (0.997–1.001)	0.435
mPAP (mmHg)	1.026 (1.002–1.050)	**0.033**
PVR (mmHg)	1.040 (1.002–1.079)	**0.037**
ε_s_ (%)	0.963 (0.935–0.992)	**0.014**
ε_e_ (%)	0.954 (0.909–1.001)	0.053
ε_a_ (%)	0.959 (0.919–1.002)	0.059
SRs (s^−1^)	0.851 (0.474–1.531)	0.591
SRe (s^−1^)	1.745 (0.967–3.148)	0.065
SRa (s^−1^)	1.689 (1.059–2.695)	**0.028**
tRAEF (%)	0.983 (0.960–1.006)	0.141
pRAEF (%)	0.991 (0.960–1.023)	0.571
aRAEF (%)	0.989 (0.969–1.010)	0.313
RVEF (%)	0.981 (0.952–1.011)	0.211
RVEDVi (mL/m^2^)	1.008 (1.000–1.016)	0.051
RVESVi (mL/m^2^)	1.010 (1.001–1.019)	**0.033**
RVSVi (mL/m^2^)	1.003 (0.977–1.030)	0.806
RV GLS (%)	1.048 (0.975–1.127)	0.200
LVEF (%)	0.989 (0.962–1.018)	0.464
LVEDVi (mL/m^2^)	0.980 (0.962–0.997)	**0.023**
LVESVi (mL/m^2^)	0.982 (0.956–1.008)	0.172
LVSVi (mL/m^2^)	0.961 (0.931–0.992)	**0.015**

Significant *p*-values (*p* < 0.05) are indicated in bold

*mPAP* mean pulmonary arterial pressure, *PVR* pulmonary vascular resistance, *WHO FC* World Health Organization functional classification, *6MWD* 6-min walk distance, *BNP* brain natriuretic peptide, *ε*_*s*_ total strain, *ε*_*e*_ passive strain, *ε*_*a*_ active strain, *SRs* total strain rate, *SRe* passive strain rate, *SRa* active strain rate, *tRAEF* total RA emptying fraction, *pRAEF* passive RA emptying fraction, *aRAEF* active RA emptying fraction, *RVEF* right ventricular ejection fraction, *RVEDVi* right ventricular end-diastolic volume index, *RVESVi* right ventricular end-systolic volume index, *RVSVi* right ventricular stroke volume index, *RV GLS* right ventricular global longitudinal peak strain, *LVEF* left ventricular ejection fraction, *LVEDVi* left ventricular end-diastolic volume index, *LVESVi* left ventricular end-systolic volume index, *LVSVi* left ventricular stroke volume index

## Discussion

To our knowledge, this is the first study to investigate RA functional characteristics before and after RVSD using CMR and to explore the long-term prognostic value of RA strain. We assessed RA strain in patients with precapillary PH, focusing on morphological and functional changes in the RA in the early stage before RVSD and its prognostic value, with the following findings. Compared with HCs, RA reservoir and conduit functions significantly decreased in PH-nonRVSD patients, whereas there were no significant morphological changes in either the RA or RV. More importantly, the above parameters were significantly correlated with RVEF, RV GLS, PVR, BNP, CI, and the 6MWD. The detection performance of RA conduit function for PH-nonRVSD was superior to that of RV GLS and RVEF. In PH-RVSD patients, RA function deteriorates, particularly the booster pump function, indicating potential disease progression. RA reservoir function was an independent predictor of long-term mortality outcomes in PH patients.

In PH patients, RVSD represents more advanced clinical characteristics and predicts severe events and poorer outcomes [4, 16]. However, few studies have investigated the characteristics of RA function before and after RVSD. In our study, stratification of PH patients based on RVSD status revealed that impaired RA reservoir (ε_s_ and SR_s_) and conduit (ε_e_ and SR_e_) functions were already present before RVSD onset. Additionally, we found that ε_s_ and ε_e_ were correlated with RV GLS, RVEF, and PVR. The detection performance of ε_e_ in differentiating PH-nonRVSD patients from HCs was superior to that of RV GLS and RVEF. The results indicate that changes in the RA reservoir and conduit functions reflect early functional impairment and afterload increase in the right heart. These could be more sensitive indicators for detecting impaired RV function in PH patients before RVSD. As RV pressure increases with elevated RV afterload, the pressure gradient between the RV and RA decreases, leading to RV diastolic dysfunction. This may underlie the observed impairment conduit function [12]. Due to chronic elevation in RV pressure, the RA wall is often stretched and stiffened [17]. In this situation, it becomes difficult for the RA to increase atrial filling.

In our study, RA booster pump function (ε_a_ and SR_a_) was preserved in PH-nonRVSD patients but declined in PH-RVSD patients. These findings suggest that impaired RA booster pump function may be a critical indicator of RVSD in precapillary PH patients. Moreover, RA reservoir and conduit functions were more severely affected, and RV GLS was further reduced in PH-RVSD patients. The booster pump function could maintain and compensate for the impaired reservoir and conduit functions to supplement RV filling before RVSD. With disease progression and RVSD development, this mechanism becomes decompensated. Leng et al also reported that PAH patients with decompensated RV function exhibited significantly impaired RA function across all three phases compared with those with compensated RV function [18]. Moreover, RV morphological parameters were abnormal in PH patients with RVSD. Therefore, both RV and RA function are further compromised, and the morphology of both the RV and RA also changes as PH progresses to RVSD, potentially leading to RA‒RV uncoupling.

The decline in RA reservoir function, as measured by ε_s_, was progressive across HCs, PH-nonRVSD patients, and PH-RVSD patients. Similarly, RA conduit function, as assessed by ε_e_ and SR_e_, also showed a downward trend. We also found that RA morphology parameters (RAV_max_, RAV_pre_, and RAV_min_) were indistinctive between PH-nonRVSD patients and HCs but were significantly increased in PH-RVSD patients. These results indicate that RA functional impairment precedes morphological changes. Querejeta Roca et al [15] also found that ε_s_ was significantly reduced in PAH patients with normal RA size compared with HCs, consistent with our results. They also reported that RA reservoir and conduit functions were impaired in PAH, independent of RA size and pressure levels, and might reflect RV failure and overload. Additionally, our study demonstrated that pRAEF was reduced before RVSD and further declined in RVSD patients, and pRAEF was sensitive for detecting PH-nonRVSD patients. The tRAEF was decreased only in patients with RVSD; no significant differences were found among the three groups. Therefore, monitoring changes in RA strain function is more sensitive than assessing RA morphology and RAEF in precapillary PH.

Right heart function, including RA function, is used to predict the prognosis and guide risk stratification in PH patients [19, 20]. Vos et al reported that RA reservoir and conduit functions were predictors of all-cause mortality, with a median follow-up of 37 months [21]. Another study demonstrated that RA reservoir, conduit, and active contraction functions were independent predictors of mortality and hospitalization, with a mean follow-up of 36 months [22]. Leng et al found that RA passive function was the best predictor of composite adverse clinical outcomes, with a follow-up of 24 months [18]. We conducted long-term follow-up with a median follow-up of 97 months and found that impaired RA reservoir function was independently associated with all-cause mortality. As PH progresses, increased RV afterload and maladaptive RV remodeling and dysfunction are accompanied by RA remodeling.

The RA wall becomes stiffened due to chronic elevation of RV pressure, making it difficult for the RA to stretch and serve as a reservoir for systemic venous return. These findings suggest that a decline in RA reservoir function is a sensitive non-invasive method and a valuable indicator of long-term prognosis in PH patients. The treatment goal for PH is to maintain or achieve a low-risk status, which is closely associated with reduced mortality [13]. Yamasaki et al reported that impaired reservoir and conduit functions were markedly improved by balloon pulmonary angioplasty (BPA) in CTEPH patients [23]. BPA improved pulmonary haemodynamics, exercise tolerance, and clinical outcomes. This improvement in RA function might have contributed to improvements in the clinical outcomes of patients with CTEPH. PAH patients with worsening RA reservoir function have significantly poorer prognoses than those with stable or improved RA reservoir function [24]. Therefore, PAH patients may exhibit differing RA functional response patterns to PAH therapy. Improvements in RA function may be driven by reductions in afterload, RV remodeling, and RV dysfunction. Whether RA functional recovery following treatment contributes to meaningful clinical recovery warrants further investigation in future studies.

The correlation analysis revealed that ε_s_, ε_e_, and ε_a_ were not correlated with mRAP. However, Leng et al reported the opposite result, showing that ε_s_ was inversely correlated with RAP. Compared with the participants in their study, the PH patients in our study had significantly lower mRAP, smaller RA sizes, and lower WHO-FC levels. In particular, only 17% of patients in our cohort had an mRAP between 10 and 12 mmHg, whereas 28% of patients had an mRAP greater than or equal to 10 mmHg in the study of Leng et al [18]. This discrepancy may be attributed to differences in disease stage among the included participants. In addition, Wright et al [9] reported no significant correlation between RA strain values (ε_s,_ ε_e_, and ε_a_) obtained from echocardiography and RAP (median 8 mmHg). Therefore, further studies are needed to clarify the correlation between RA strain and invasive RAP, and to explore how RA function changes at different stages of precapillary PH, particularly from the perspective of RAP.

Our study had several limitations. First, it was a single-center study, and a larger-scale multicenter study is needed. Second, RA strain was analyzed using a module validated for ventricular analysis, as no vendor-independent commercial software for RA strain analysis was available at the time. However, this method has been widely recognized and used in previous studies [11, 25, 26], and we demonstrated high intra- and interobserver reproducibility. Finally, the interval between CMR and RHC in the included patients was 6 (1–12) days, which may have influenced the results. We plan to minimize this interval in future studies to improve data precision.

## Conclusions

In conclusion, RA functional impairment is a promising indicator for early diagnosis and progression monitoring in precapillary PH patients, providing comprehensive information for risk stratification and long-term prognosis.

## Supplementary information

ELECTRONIC SUPPLEMENTARY MATERIAL

## Data Availability

The datasets and material generated or analyzed during the study are available from the corresponding author on reasonable request.

## Abbreviations

aRAEF: Active right atrial emptying fraction
CMR-FT: Cardiac magnetic resonance feature tracking
GLS: Global longitudinal peak strain
HCs: Healthy controls
PH-nonRVSD: Pulmonary hypertension patient without right ventricular systolic dysfunction
PH-RVSD: Pulmonary hypertension patient with right ventricular systolic dysfunction
pRAEF: Passive right atrial emptying fraction
RA: Right atrium
RAV_max_: Right atrial maximal volume
RAV_min_: Right atrial minimal volume
RAV_pre_: Right atrial pre-emptying volume
RV: Right ventricle
RVEF: Right ventricular ejection fraction
RVSD: Right ventricle systolic dysfunction
SR_a_: Active strain rate
SR_e_: Passive strain rate
SR_s_: Total strain rate
tRAEF: Total right atrial emptying fraction
ε_a_: Active strain
ε_e_: Passive strain
ε_s_: Total strain
