# Correction: Impaired right atrial function preceding right ventricular systolic dysfunction: clinical utility and long-term prognostic value in pulmonary hypertension

**DOI:** 10.1186/s13244-025-02071-w

**Published:** 2025-11-27

**Authors:** Fan Yang, Yan Yan, Wang Jiang, Zhouming Wang, Caixin Wu, Qian Wu, Yuanlin Deng, Yamin Du, Zhenwen Yang, Zhang Zhang, Dong Li

**Affiliations:** 1 Department of Radiology, Tianjin Medical University General Hospital, Tianjin, China; 2 Department of Radiology, Tianjin Key Lab of Functional Imaging & Tianjin Institute of Radiology, Tianjin Medical University General Hospital, Tianjin, China; 3 Department of Cardiology, Tianjin Medical University General Hospital, Tianjin, China; 4 Department of Radiology, Tangshan Maternal and Child Health Hospital, Hebei, China

**Correction to: Insights into Imaging**

10.1186/s13244-025-01996-6published online 04 June 2025.

The Graphic Abstract in the original article contained an incorrect diagram:

**Figure:**
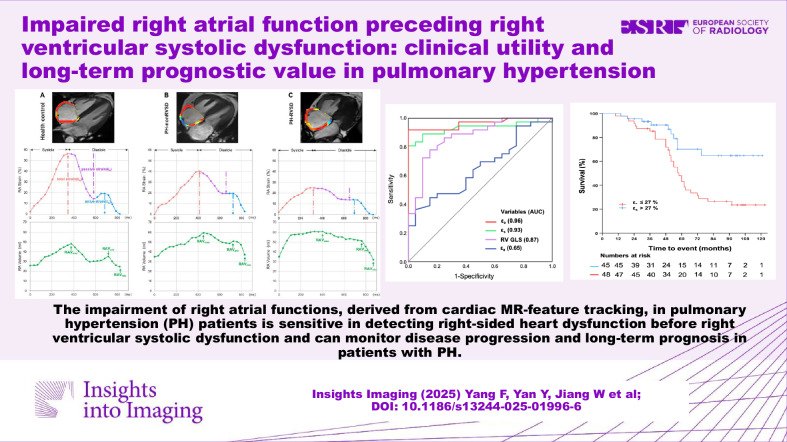

**Figure:**
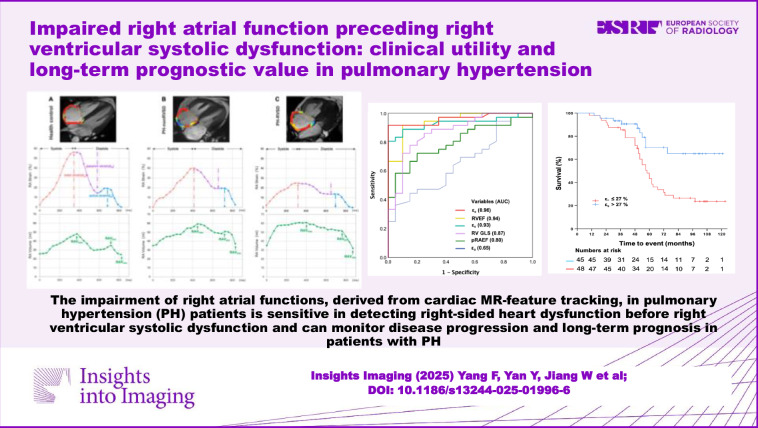

The corrected Graphic Abstract can be seen below:

The original article has been corrected.

