# Supplementary material for: Impaired right atrial function preceding right ventricular systolic dysfunction: clinical utility and long-term prognostic value in pulmonary hypertension
Source: Insights Imaging. 2025 Jun 4;16:115. doi: 10.1186/s13244-025-01996-6 (PMC12137862; doi:10.1186/s13244-025-01996-6)
Supplement: Supplementary file 1 — ELECTRONIC SUPPLEMENTARY MATERIAL [file 13244_2025_1996_MOESM1_ESM.pdf]

Impaired right atrial function preceding right ventricular systolic dysfunction: Clinical utility and long-term prognostic value in pulmonary hypertension

ELECTRONIC SUPPLEMENTARY MATERIAL

CMR acquisition and analysis

Typical parameters for cine images of two scanners were as follows: repetition time, 3.2-3.4msec; echo time,1.4-1.5msec; field of view, 350 × 350 mm^2; matrix, 224 × 224; flip angle, 45°; and number of frames, 25 per cardiac cycle. The slice thickness was 8 mm for short-axis images and 6 mm for four-chamber images without the gap between slices.

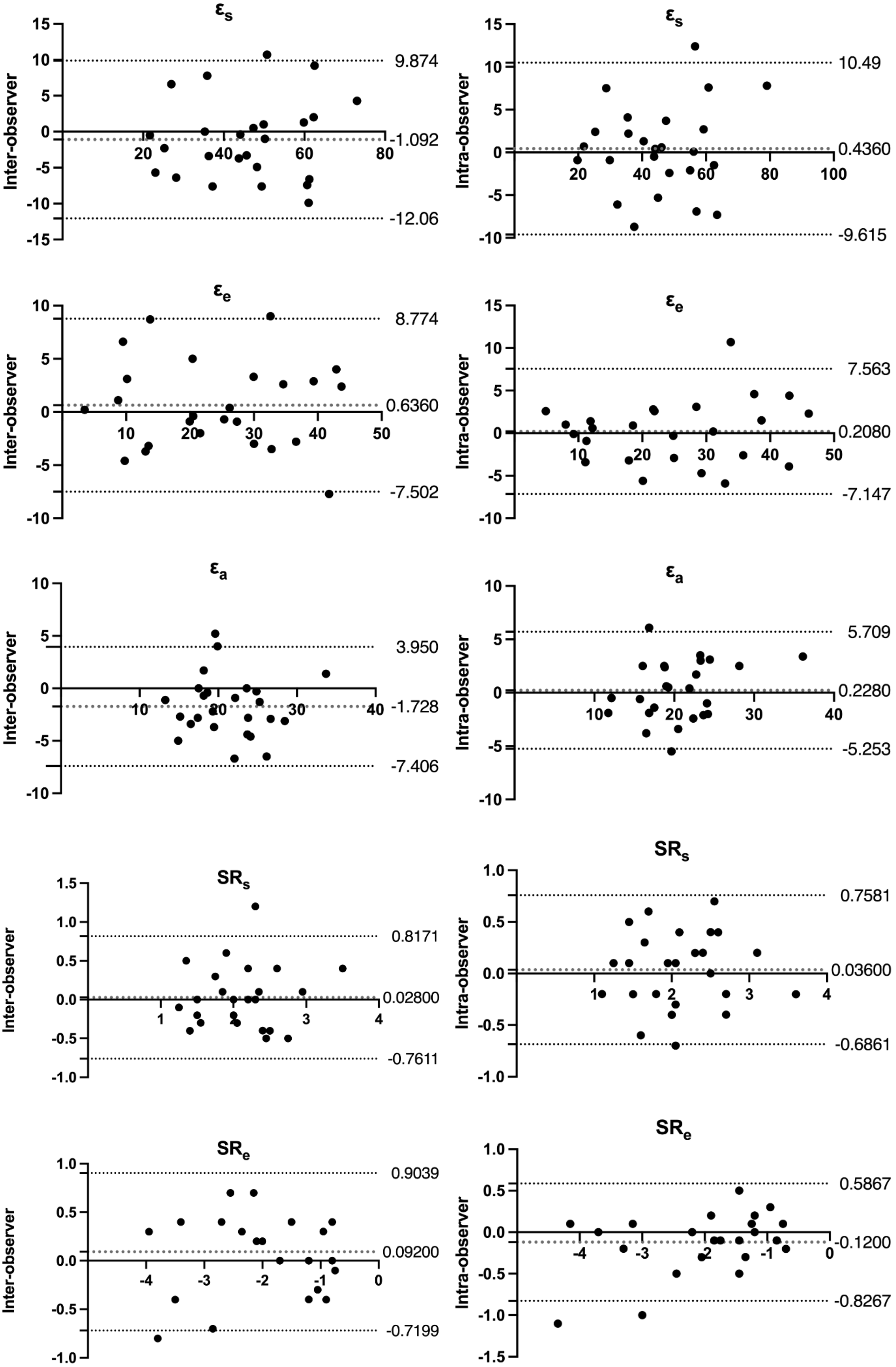

Insights Imaging (2025) Yang F, Yan Y, Jiang W, et al.

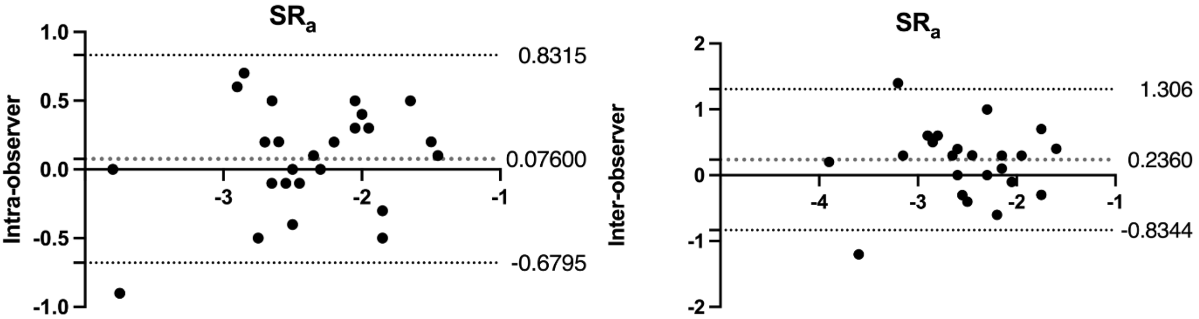

Figure S1. Bland-Altman plots showed intra- and inter-observer variability of RA strain and strain rate parameters.

Table S1. Right atrium (RA) characteristics of PH patients after adjusting comorbidities including hypertension and diabetes mellitus.

Variables	PH-nonRVSD (n = 36)	PH-RVSD (n = 60)	*P* Value
RA strain (%)			
ε_s	34 (28-43)	21 (14-29)	<0.001
ε_e	16 (12–21)	10 (7–16)	<0.001
ε_a	19 ± 7	10 ± 7†††	<0.001
RA strain rate (sec^{-1})			
SR_s	1.7 (1.1 - 2.2)	1.1 (0.8-1.4)	<0.001
SR_e	-1.3 (-1.7 to -0.8)	-0.7 (-1.0 to -0.5)	<0.001
SR_a	-2.2 (-2.7 to -1.6)	-1.4 (-1.7 to -0.9)	<0.001
RA volume index (mL/m^2)			
RAV_{max} index	39 (32–48)	48 (36–74)	0.015
RAV_{pre} index	31(24–41)	45(33–68)	0.002
RAV_{min} index	18(15–27)	29(20–42)	<0.001
RA ejection fraction (%)			
tRAEF	46 ± 13	40 ± 12	0.01
pRAEF	15 ± 11	11 ± 8	0.018
aRAEF	36 ± 13	33 ± 13	0.137

RA, right atrial, PH-nonRVSD, pulmonary hypertension patients without right ventricular systolic dysfunction; PH-RVSD, pulmonary hypertension patients with right ventricular systolic dysfunction; ε_s, total strain; ε_e, passive strain; ε_a, active strain; SR_s, total strain rate; SR_e, passive strain rate; SR_a, active strain rate; RAV_{max}, RA maximal volume; RAV_{pre}, RA pre-emptying volume; RAV_{min}, RA minimal volume; tRAEF, total RA emptying fraction; pRAEF, passive RA emptying fraction; aRAEF, active RA emptying fraction.

Table S2. Intra- and inter-observer variability of strain and strain rate parameters.

Variables	Intra-observer		Inter-observer	
	ICC	95%CI	ICC	95%CI
ε_s, %	0.94	0.88–0.98	0.93	0.84–0.97
ε_e, %	0.96	0.90–0.98	0.94	0.88–0.97
ε_a, %	0.87	0.72–0.94	0.79	0.50–0.91
SR_s, sec^{-1}	0.83	0.65–0.92	0.78	0.56–0.90
SR_e, sec^{-1}	0.94	0.87–0.97	0.92	0.82–0.96
SR_a, sec^{-1}	0.80	0.61–0.91	0.73	0.48–0.87

ICC, intraclass correlation coefficient; CI, confidence interval; ε_s, total strain; ε_e, passive strain; ε_a, active strain; SR_s, total strain rate; SR_e, passive strain rate; SR_a, active strain rate.

Table S3. Receiver operating characteristic (ROC) analysis of right atrium and right ventricle function parameters to differentiate pulmonary hypertension patients without right ventricle systolic dysfunction (PH-nonRVSD) from health controls.

Variables	AUC(95% CI)	Sensitivity	Specificity	*P* value
ε_e	0.96(0.91-1.00)	0.92	1.00	<0.001
ε_s	0.93(0.86-1.00)	0.81	1.00	<0.001
RVEF	0.94(0.89-1.00)	0.92	0.90	<0.001
RVGLS	0.87(0.77-0.97)	0.78	0.85	<0.001
pRAEF	0.80(0.69-0.91)	0.58	0.95	<0.001
ε_a	0.65(0.51-0.80)	0.36	0.95	0.061

AUC, area under the curve; CI, confidence interval; RVEF, right ventricular ejection fraction; RVGLS, right ventricular global longitudinal peak strain; ε_s, total strain; ε_e, passive strain; ε_a, active strain; pRAEF, passive right atrial emptying fraction.

Table S4. Predictors of mortality with multivariate cox proportional hazard regression analysis in patients with PH.

	Multivariate analysis	
Variables	HR (95%CI)	P Value
mPAP, mmHg		0.090
PVR, mmHg		0.237
ε_s, %	0.963 (0.935-0.992)	**0.014**
SRa, sec-1		0.542
RVESVi, mL/m^2		0.435
LVEDVi, mL/m^2		0.153
LVSVi, mL/m^2		0.104

Significant *P* -values (P < 0.05) are indicated in bold.

mPAP, mean pulmonary arterial pressure; PVR, pulmonary vascular resistance; ε_s, total strain; SRa, active strain rate; RVESVi, right ventricular end-systolic volume index; LVEDVi, left ventricular end-diastolic volume index; LVSVi, left ventricular stroke volume index.
